# Effects of Oral Contraceptive Androgenicity on Visuospatial and Social-Emotional Cognition: A Prospective Observational Trial

**DOI:** 10.3390/brainsci10040194

**Published:** 2020-03-25

**Authors:** Caroline Gurvich, Annabelle M. Warren, Roisin Worsley, Abdul-Rahman Hudaib, Natalie Thomas, Jayashri Kulkarni

**Affiliations:** Monash Alfred Psychiatry Research Centre, Central Clinical School, Monash University and The Alfred Hospital, Melbourne, VIC 3004, Australia; annabellemwarren@gmail.com (A.M.W.); rosie.worsley@gmail.com (R.W.); abdul-rahman.hudaib@monash.edu (A.-R.H.); natalie.thomas@monash.edu (N.T.); jayashri.kulkarni@monash.edu (J.K.)

**Keywords:** oral contraceptive, cognition, progestin, androgenicity, estrogen, progesterone

## Abstract

Oral contraceptives (OCs) containing estrogen and progesterone analogues are widely used amongst reproductive-aged women, but their neurocognitive impact is poorly understood. Preliminary studies suggest that OCs improve verbal memory and that OCs with greater androgenic activity may improve visuospatial ability. We sought to explore the cognitive impact of OCs by assessing performance of OC users at different stages of the OC cycle, and comparing this performance between users of different OC formulations according to known androgenic activity. We conducted a prospective, observational trial of OC users, evaluating cognitive performance with CogState software on two occasions: days 7–10 of active hormonal pill phase, and days 3–5 of the inactive pill phase (coinciding with the withdrawal bleed resembling menstruation). Thirty-five OC users (18 taking androgenic formulations, 17 taking anti-androgenic) were assessed. Analysis by androgenic activity showed superior performance by users of androgenic OCs, as compared to anti-androgenic OCs, in visuospatial ability and facial affect discrimination tasks. A growing understanding of cognitive effects of OC progestin androgenicity may have implications in choice of OC formulation for individuals and in future OC development.

## 1. Introduction

Oral contraceptives (OCs) are the most commonly prescribed medication in women of reproductive age to avoid unintended pregnancies and to alleviate menstrual pain, premenstrual syndrome, acne, and heavy bleeding [1]. The psychological effects of OCs can include depression and adverse mood effects [2]. The cognitive effects of OCs are not well studied [3].

An established body of research shows that estrogens, progesterones, and androgens can influence cognitive processes [4,5,6,7], with these reproductive hormones thought to contribute to differences in cognitive performance that have been observed between sexes, as well as enhancement in cognition associated with hormone-based therapies [8]. Several studies describe verbal skills as female-favoring, visuospatial ability as male-favoring, and social-emotional cognition as female-favoring [4,9,10,11,12], although much contemporary research also recognizes a role for socio-cultural factors in these reported sex differences [13]. In females, cognitive changes, including impairments in memory, executive functioning, and verbal fluency, have been reported during pregnancy [14,15] and during the menopause [16], with endogenous hormone flux implicated in these cognitive changes. The menstrual cycle also provides a convenient model to examine the influence of endogenous sex hormones on cognitive functioning. The underlying hypothesis for most menstrual cycle studies is that performance on verbal tasks is improved during the luteal phase, when estradiol levels are high (following pre-ovulatory peak) and progesterone is maximal; whilst performance on visuospatial tasks is optimal during the menstrual phase when hormone levels are low [17,18]. Menstrual cycle variations have also been reported in emotion processing in healthy women as well as women with premenstrual disorders and these emotion processing fluctuations appear to be associated with progesterone levels [19]. In their comprehensive review of the topic, Sundstrom Poromaa and Gingnell [20] concluded that menstrual-cycle-related variations in “sexually dimorphic” cognitive tasks are small and difficult to replicate; however, emotion-related changes are more consistently found and appear to be associated with progesterone. 

At the molecular level, established neuromodulatory, neuroprotective, and neuroproliferative effects of hormones both in vitro and in vivo provide potential mechanisms for such hormone-dependent effects on cognition [6,7,21]. A substantial body of research has established that ovarian hormones can induce spinogenesis and synaptogenesis in brain regions relevant to cognition, primarily the prefrontal cortex and hippocampus [22]. Estrogens, progesterones, and androgens work via genomic actions, mediating gene transcription through nuclear receptor functions as well as via fast-acting, nongenomic activity, whereby estrogen and progestin act via extranuclear and membrane-bound receptors, altering downstream intracellular signaling pathways that affect synaptic function [22,23]. Neurosteroids, including estrogen and androgens, have also been demonstrated to be synthesized de novo in brain regions, allowing for rapid action at the level of the central nervous system, affecting neural activity and cognitive processes [24].

Combined oral contraceptive formulations generally follow a 28-day cycle of 21–24 ‘active’ pills containing synthetic analogues of estrogen and progesterone, followed by 4–7 days of hormone-free inactive pills leading to a withdrawal bleed resembling menstruation. Administration of exogenous estrogen and progestins provides negative feedback to the hypothalamic–pituitary–gonadal axis, leading to a downregulation of endogenous sex hormone availability in OC users. Refinements in OC formulation have led to the current diversity of OCs, with varied side-effect profiles partly relating to the androgenic activity of the progestin component [25]. Most current formulations contain ethinylestradiol as the estrogen component (with the exception of two newer formulations which contain estradiol and estradiol valerate), with a variable progestin component [26]. Older first- and second-generation OCs contain progestins structurally related to testosterone (e.g., levonorgestrel, norethisterone), with corresponding androgenic side-effect profiles. Newer third- and fourth-generation OCs contain progestins more closely resembling progesterone (e.g., cyproterone acetate, drospirenone) with neutral androgenicity or even anti-androgenic effects [27]. 

The cognitive impact of OCs has been investigated by approximately 22 studies over the past 50 years of use, but recent systematic reviews by our group and others show that a consensus remains elusive [3,28]. Inconsistent methodology with inadequate cognitive batteries, irregular cyclic timing, and limited power may contribute to the heterogeneity of findings. The most reliable outcome in the literature is of improvement in verbal memory with OC use, both compared with naturally-cycling women [29] and in the active versus inactive OC phase [30]. This is likely to be attributable to the effect of ethinylestradiol, as estrogens are documented to improve verbal performance [4,17,31], although a clear association between estradiol dose and verbal memory performance has not been documented (and one study failed to find an association between estradiol dose and other verbal abilities [32]). Some studies have found no significant impact of OC use on verbal memory both in comparison to OC nonusers and across the OC cycle [33,34].

Studies investigating associations between OC use and visuospatial abilities have mixed results. Several studies have reported no differences in mental rotation task performance (in terms of accuracy) between OC users and naturally cycling females [30,35,36], and some studies have suggested that OC use is associated with superior mental rotation abilities relative to naturally cycling females [32]. Wharton et al. [37] were the first to analyze the cognitive impact of OCs in this area according to progestin androgenicity, finding that anti-androgenic OCs containing drospirenone impaired visuospatial ability (mental rotation), whereas androgenic OCs improved visuospatial ability relative to the performance of OC nonusers. Wharton proposed that the differential effects of progestins, leading to both improvement and impairment in this male-favoring domain, may mask one another when OCs are examined collectively, which may explain the discrepancy in previous findings. Analysis by androgenicity has been replicated in one other study, which found nonsignificant trends in relation to mental rotation [36], and has not been extended to other cognitive domains such as social-emotional cognition. 

Previous research linking OC use to processing of social-emotional information has been mixed, although a recent review proposed that emotional processes are influenced by OC use [28]. A small body of work has demonstrated that OC users have a blunted salivary cortisol responsivity, relative to OC nonusers, following laboratory stressors (e.g., the Trier Social Stress Test (TSST) psychosocial stress paradigm) [38,39]. OC users demonstrate a pattern whereby salivary cortisol levels remain high, relative to OC nonusers, across a control and stressor (TSST) condition (i.e., a lack of a cortisol response following the stressor), and this effect is maintained during both the active and inactive pill phases [40]. In relation to emotion recognition, OC users have been demonstrated to have less accurate recognition of negative and complex emotional expressions as compared to nonusers in some studies [41,42,43] but not others [44]. OC users have also been demonstrated to have better affective responsiveness (a measure of empathy) as compared to nonusers and users in the inactive pill phase [44]. Various factors may contribute to the variations in findings, including methodological design differences (i.e., cross-sectional versus longitudinal designs) as well as differences in OC pill types across studies.

Our study aimed to investigate the cognitive impact of OC androgenicity across a range of cognitive domains, including domains hypothesized to be sensitive to estrogens, progesterones, and androgens, such as verbal memory and visuospatial abilities. Given the preliminary evidence for a differential impact of OC androgenicity in visuospatial ability, we hypothesized that progestin androgenicity group differences would be observed in these domains. We hypothesized that differences may also be seen in other domains with known sex differences or menstrual cycle fluctuation, such as social-emotional ability. The underexplored effects of progestin androgenicity may help explain the controversy surrounding the cognitive impact of oral contraceptives to date.

## 2. Materials and Methods

We conducted a prospective, observational trial of healthy female OC-users, evaluating cognitive performance on two occasions: days 7–10 of active hormonal pill phase and days 3–5 of the inactive phase (coinciding with the withdrawal bleed). The project received ethics approval from the Alfred Hospital Ethics Committee (46-09) and Monash University Human Research Ethics Committee (CF09/1895-2009001079). 

Inclusion criteria included current OC use, age over 18 years, fluency in English, and the ability to provide informed consent and attend two face-to-face interviews. Exclusion criteria included menopause, pregnancy or breastfeeding within the past 12 months, medical contraindications to OC use (including thromboembolic disease, cardiovascular disease, and cancer), and any history of neurological or cognitive impairment. Of 79 volunteers who responded to study advertisements, 21 declined to participate, 20 were excluded (nine for logistic reasons, eight not currently taking OCs, two used active pills continuously, and one was postpartum). Thirty-eight participants entered the study, three of whom withdrew prior to completion.

Following written informed consent, demographic information was collected, including age, body mass index (BMI), ethnicity, handedness, education, and income; collected information about OC use included duration, adherence, and rates of adverse effects. Evaluation of premenstrual symptom burden was assessed using the Menstrual Distress Questionnaire [45]. Mood was evaluated using the Beck Depression Inventory (BDI) [46], the Center for Epidemiological Studies Depression Scale—Revised (CESD-R) [47], and the Profile of Mood States (POMS) [48] at both visits. 

Cognitive testing was performed using ‘CogState Research’ software (Cogstate Ltd., Melbourne, VIC, Australia). CogState has been validated in healthy populations [49,50] and shown to be robust against practice effects (i.e., improvement with repeated exposure) [51]. 

Participants were presented the program on a MacBook laptop computer. Completion of the battery took approximately 45 min and included assessment of verbal memory and recall, visual memory and recall, attention, executive function, psychomotor speed, and social-emotional ability via facial affect recognition, as summarized in Table 1. Participants completed all tasks in the same order on both testing occasions. All tasks were presented initially in ‘training’ form before assessment.

OCs were categorized according to androgenic or anti-androgenic activity based on classifications established in current literature [52]. Order of testing visits was determined according to stage of cycle at time of enrolment. Of 35 participants, 18 commenced testing during the ‘inactive’ phase (eight androgenic OC users, 10 anti-androgenic), and 17 commenced testing during the ‘active’ pill phase (10 androgenic OC users, seven anti-androgenic). 

The ‘inactive’ interview was arranged to coincide with the withdrawal bleed, days 1–3 of menstrual cycle, typically days 3–5 of inactive pill intake, with the exception of two participants taking Zoely and Qlaira who were testing during their shorter inactive pill window. This enabled maximum washout period while maintaining endogenous hormone suppression. The ‘active’ interview was timed to coincide with days 14–16 of the menstrual cycle, following continuous administration of at least seven days of active pills. There was a two-week interval between sessions. If a participant was unavailable to complete the second interview two weeks after the first, the follow-up interview was delayed until the subsequent menstrual cycle, which occurred for nine participants (six androgenic OC users, three anti-androgenic).

Interviews were conducted in a private room to ensure confidentiality and minimize distraction. The two interviews were conducted at a similar time of day to eliminate the potential impact of diurnal variation. Participants were instructed to adhere to their normal dietary intake during the study period, including their typical pattern of caffeine and alcohol consumption. All participants confirmed they were not intoxicated at the time of testing.

**Statistical analyses:** Variables OC class (anti-androgenic vs. androgenic) and pill phase (active vs. inactive) were factors. The dependent variables were cognitive task scores generated by the CogState program (incorporating automatic transformation of some variables, see Table 1). There were two experimental units in the study; the first one was the subject participant unit where cognitive scores collected from the same participant on two different pill phases were correlated. The second unit was the pill class where observed cognitive scores for the two groups were unique (17 and 18 subjects for anti-androgenic and androgenic groups, respectively). To evaluate the effects of pill class and pill phase on cognitive task scores, we used Mixed and GLIMMIX SAS procedures (SAS 9.4, SAS, Cary, NC), to fit random effects parametric models (generalized Poisson or beta or normal distribution). Each dependent variable was analyzed in a separate model where its error distribution was appropriately specified. The subject effect, pill phase, and pill phase × subject interaction, were written in two random/repeated option statements. Due to small sample size and the variance differences between the two groups, the denominator degrees of freedom used the method of Kenward–Roger correction. Alpha level was set at 0.05 for two-tailed t distributions. Further, *p* values were adjusted for multiplicity using the false discovery rate (FDR) method.

In addition, further exploratory analysis was performed to determine the impact of other variables (e.g., age, ethinylestradiol dose, mood score) on cognitive performance in the form of correlations across the cohort as a whole and separately within each progestin group.

## 3. Results

Our final sample included 35 females with mean age 22.9 years and mean BMI 21.31 kg/m^2^. Twenty-five participants racially identified as white, with the remaining ten of mixed or Asian/Indian ethnicity. The majority were university students (91%). 

Comparable numbers of participants were taking ‘androgenic’ (first and second generation) OC formulations as were taking ‘anti-androgenic’ (third and fourth generation) brands, 18 and 17 women, respectively (Table 2). Androgenic and anti-androgenic OC user groups did not differ significantly on demographic variables, premenstrual symptom burden, mood, duration of OC use, adherence, or rates of adverse effects experienced (Table 3).

Table 4 shows the mean values for each cognitive task by pill phase and pill class, and the results of the Mixed and GLIMMIX SAS procedures are shown in Table 5. We did not find evidence against the respective null hypotheses of no differences between the two pill phases, active and inactive, for any cognitive task. In relation to pill class, as seen in Table 5, for the visuospatial tasks, there were fewer task errors (maze, maze recall and shapes) for participants in the androgenic pill class as compared to anti-androgenic class. For the maze recall task, there was some evidence against the null hypothesis of no task performance differences between the two classes (higher log error count of 0.4358 for the anti-androgenic class, adjusted *p* = 0.04). Similarly, there was evidence against the null hypothesis that social-emotional task accuracy proportions were equal for both classes (adjusted *p* = 0.04), with the ratio (accuracy proportion) for anti-androgenic pill class participants being *exp* (−0.6346) *100% = 53% (less) than for the androgenic class participants. 

The adjusted *p*-values for verbal, executive function, and psychomotor tasks support the conclusion there was weak or no evidence against the respective null hypotheses of no differences between the two pill classes. The one- and two-back task scores were not fitted in the random effects model because the algorithm failed to obtain the minimum variance quadratic unbiased estimates as starting values for the covariance parameters. Nevertheless, Table 4 shows the differences in performance indices between the two pill classes for the two attention/memory domain tasks, and the mean scores are similar at a descriptive level for pill phase and pill class.

## 4. Discussion and Conclusions

The key finding from this study was a significant difference in visuospatial and social-emotional performance in androgenic compared to anti-androgenic OC users. We did not observe differences between active and inactive pill phases in any cognitive domain.

The observation of improved visuospatial performance in users of OCs containing androgenic progestins throughout the cycle confirms and expands previous studies [36,37]. Our study showed a similar pattern of improved visuospatial performance in OC users with androgenic progestins compared to anti-androgenic OC users across all three visuospatial tasks—mazes, shapes, and maze recall. However, the difference only reached statistical significance for the maze recall task. The limited previous studies in this area have suggested an advantage for androgenic progestins in the area of mental rotation [36,37]. Our findings expand this to include visuospatial learning and recall, which is also a cognitive domain where better performance has been linked to endogenous [53] and exogenous testosterone [54,55] in females. Furthermore, visuospatial learning has been linked to hippocampal androgen receptor activation in animal models [56]. Therefore, as our findings suggest, it is possible that progestins classified as androgenic and structurally related to testosterone are more likely to enhance visuospatial abilities. 

In addition, users of androgenic OCs also performed more accurately than anti-androgenic OC users on the social-emotional task that assessed facial emotion recognition/discrimination. An effect of androgenicity on facial emotion processing is without precedent in the literature. Facial emotion recognition/processing has been associated with a female advantage [57], and a role for sex hormones has been implicated [58]. The few existing studies that have specifically examined facial emotion recognition and OC use have reported both impaired emotion recognition in OC users versus nonusers [41,42,43] as well as no difference between OC users and nonusers [44]. It has been suggested that OC use may impair the capacity to recognize complex emotional expressions [43]. OC users have previously demonstrated a stronger neural response to faces with angry and ambiguous expressions in the right fusiform face area when compared to freely cycling women [59], and one previous study reported that androgenic and anti-androgenic OCs differentially modulate grey matter volume in regions associated with neutral face recognition [60]. Neutral face recognition has also been demonstrated to be more accurate in users of anti-androgenic OCs compared to androgenic OC users [60]. Collectively, findings suggest that OC use influences facial emotion recognition, and, while neutral face recognition may be superior in anti-androgenic OC users [60], our findings suggest that emotional face recognition is more accurate in users of androgenic OCs. Of relevance, three clinical studies involving exogenous testosterone administration have demonstrated associations between higher testosterone levels and increased brain activity during facial emotion recognition in females [61,62,63]. Hence it is possible that androgenic progestins, that are structurally more related to testosterone, have the capacity to enhance facial emotion recognition. Further research breaking down recognition and processing of different emotion types is needed to better understand how OC use and progestin types influence facial emotion processing.

Results in the domains of verbal learning and memory, attention, executive function, and psychomotor ability demonstrated no effect of OC androgenicity. This is in line with our hypotheses as well as an absence of previous research indicating that androgenicity might affect these cognitive processes.

Limitations of our study included the lack of a control group of OC nonusers and the small sample size representing a limited subset of OC users in that all were tertiary-educated and therefore cognitively high-functioning. Our study design did not allow assessment of the effect of duration of OC use. The addition of serum hormone measurements and neuroimaging would have provided further insight.

Future research in this field may look to appraise cognition both on and off OCs to compare function in the same individuals under endogenous and exogenous hormonal conditions and may include increased sample sizes to explore newer OC formulations containing estradiol and estradiol valerate. Research regarding the impact of duration of OC use may also be informative. 

Understanding cognitive effects of OCs and progestin androgenicity may have implications in choice of OC formulation for individual women and in future OC development, as well as contributing to the growing knowledge base linking sex hormones and cognition. We acknowledge that the cognitive changes we observed are likely to be clinically subtle; however, it remains possible that OC use may still have an impact on cognition for some women. The influence of exogenous hormones on cognition is an area of growing interest. The impact of OCs on cognitive performance is poorly understood among medical professionals and patients alike. As evidence accumulates, it is important to facilitate informed contraceptive decision-making. 

## Figures and Tables

**Table 1:** List of CogState cognitive evaluation software tasks, including description of task, number of trials performed, output variable, and cognitive domain(s) assessed, presented in order of administration.

Name	Description	Trials	Variable	Cognitive Domain
List	A list of 12 “shopping list” items is read aloud to the participant, who is asked to repeat as many as they can remember.	3	Words correct	Verbal learning and memory
Chase Test	The participant is asked to ‘chase’ a target across a grid, by clicking on it and following it as it moves.	1	Moves per second	Visual motor function
Maze	The participant must uncover the hidden pathway through a grid from one corner to another, with five attempts.	5	Total errors	Executive function /visuospatial
Identification	Playing cards are presented one at a time. The participant is asked “Is the card red?” and is required to select ‘yes’ or ‘no’.	34	Speed (Tr) (log_10_ milliseconds)	Visual attention/ Vigilance
One Back	Playing cards are presented one at a time. The participant is asked “Is this card the same as the last one that was shown?” and must select ‘yes’ or ‘no’.	34	Accuracy (Tr) (arcsine square root of proportion correct)	Attention/ Working memory
Two Back	Playing cards are presented one by one. The participant is asked “Is this card the same as the card shown two cards ago?” and select ‘yes’ or ‘no’.	34	Accuracy (Tr) (arcsine square root of proportion correct)	Attention/ Working memory
Set-Shifting	Playing cards are presented sequentially. Participant is asked to identify a rule as to which card is ‘correct’ based on its number or color, by selecting ‘yes’ or ‘no’. An error sound indicates that the rule has changed, and they must identify the new rule and adapt their responses.	120	Accuracy (Tr) (arcsine square root of proportion correct)	Executive function
Shapes	Eight abstract objects are visible and the participant is asked to memorize their location. The objects then appear sequentially in the center of the screen, and the participant must identify where they were previously placed.	7	Total errors	Visuospatial learning and memory
Social-Emotional	The participant is asked to select the “odd one out” of four pictures of human faces or sets of eyes presented for 15 s. Some trials involve discriminating between facial affect presentations of different emotions (e.g., neutral vs. fear) and others require discriminating between intensities of facial affect for the same emotion (e.g., mild fear vs. extreme fear). There were also control items in which four sets of neutral eyes were shown depicting eye gaze direction (no emotion displayed), and one pair of eyes was looking in a different direction than the other three sets.	48	Accuracy (Tr) (arcsine square root of proportion correct)	Social Cognition
Maze Recall	The participant is asked to find the same hidden pathway presented earlier in the “Maze Test”.	1	Total errors	Visuospatial memory
List Recall	The participant is asked to list as many of the 12 items as they remember from the “List” they learnt earlier.	1	Words correct	Verbal memory

(Tr) = Transformed data variable (recommended CogState output).

**Table 2:** List of oral contraceptive medication formulations taken by the 35 participants in the study cohort, divided into androgenic and anti-androgenic groups (39).

OC Class	Progestin	Progestin Dose (μg)	Brand Name	Number (% total)	Active/Inactive Pill Days	EE Dose ^a^ (μg)
**Androgenic** (*N* = 18)	Levonorgestrel	150	Levlen	14 (40)	21/7	30
		150	Monofeme	2 (6)	21/7	30
		125	Microgynon	1 (3)	21/7	50
		100	50 Loette	1 (3)	21/7	20
**Anti-androgenic** (*N* = 17)	Cyproterone acetate	2	Estelle	4 (11)	21/7	35
		2	Laila	1 (3)	21/7	35
	Drospirenone	3	Yaz	4 (11)	21/7	20
		3	Yasmin	3 (9)	21/7	30
		3	Isabelle	2 (6)	21/7	30
	Dienogest	2	Valette	1 (3)	21/7	30
		variable ^c^	Qlaira ^b^	1 (3)	26/2	variable ^c^
	Nomegestrol acetate	2.5	Zoely	1 (3)	24/4	n/a ^d^

^a^ EE = Ethinlyestradiol. ^b^ Triphasic formulation. ^c^ Contains estradiol valerate 1–3 mg and dienogest 2–3 mg. ^d^ Contains estradiol 1.5 mg.

**Table 3:** Characteristics of study participants, including demographic variables, menstrual symptoms, mood scores, and OC use parameters, with comparison between androgenic and anti-androgenic OC user groups via chi-squared/T tests.

	Total *N* = 35	*Androgenic N = 18*	*Anti-androgenic N = 17*	*Androgenic vs. Anti-androgenic*
	*Mean (SD) or N (%)*			*p value*
**Demographics**				
**Age** (years)	22.97 (2.60)	*22.22 (1.11)*	*23.76 (3.42)*	0.09 (T)
**BMI**^a^ (kg/m^2^)	21.31 (2.14)	*21.36 (2.32)*	*21.25 (1.97)*	0.88 (T)
**Ethnicity**				0.65
Caucasian	25 (71%)	*12 (67%)*	*13 (76%)*	
Mixed race	6 (17%)	*4 (22%)*	*2 (12%)*	
Asian/Indian	4 (11%)	*2 (11%)*	*2 (12%)*	
**Handedness** (self-reported)				0.51
Right	32 (91%)	*17 (94%)*	*15 (88%)*	
Left	3 (9%)	*1 (6%)*	*2 (12%)*	
**Education**				0.79
Tertiary—current	24 (69%)	*13 (72%)*	*11 (65%)*	
Postgraduate—current	8 (23%)	*4 (22%)*	*4 (24%)*	
Postgraduate—complete	3 (9%)	*1 (6%)*	*2 (12%)*	
**Annual Household Income** ($AUD)				0.98
High (>$100,000)	8 (23%)	*4 (22%)*	*4 (24%)*	
Medium ($40,000–100,000)	15 (43%)	*8 (44%)*	*7 (41%)*	
Low (<$40,000)	12 (34%)	*6 (33%)*	*6 (35%)*	
**Symptom Evaluation**				
**Menstrual Symptoms** (MDQ Score)	7.7 (7.7)	*5.9 (5.9)*	*9.7 (8.9)*	0.16 (T)
**Mood** (average score)				
CESD-R	7.9 (6.4)	*6.8 (5.2)*	*9.0 (7.5)*	0.30 (T)
BDI ^a^	4.4 (3.9)	*3.3 (2.7)*	*5.5 (4.8)*	0.11 (T)
POMS ^a^	20.3 (26.2)	*17.4 (22.5)*	*23.6 (30.3)*	0.50 (T)
**Oral Contraceptive Use**				
**Duration current OC use**				
<6 months	8 (23%)	*3 (17%)*	*5 (29%)*	0.31
6–12 months	6 (17%)	*2 (11%)*	*4 (24%)*	
>12 months	21 (60%)	*13 (72%)*	*8 (47%)*	
**Adherence**				
Good (0–1 missed/month)	30 (86%)	*17 (94%)*	*13 (76%)*	0.13
Poor (2+ missed/month)	5 (14%)	*1 (6%)*	*4 (24%)*	
**Side effects on current OC (self-reported)**				
Physical	8 (23%)	*5 (28%)*	*3 (18%)*	0.43
Psychological	1 (3%)	*1 (6%)*	*0 (0%)*	0.37

^a^ Note missing data from BMI (*N* = 33) and BDI/POMS mood scores (*N* = 34). Abbreviations: MDQ = Menstrual Distress Questionnaire; CESD-R = Centre for Epidemiological Studies Depression Scale—Revised; BDI = Beck Depression Inventory; POMS = Profile of Mood States. (T) indicates variables where T test was used for comparison between androgenic and anti-androgenic OC users. Where not otherwise indicated, comparison was made using chi-squared tests.

**Table 4:** Mean (SD or SE) for each cognitive task by pill phase and pill class.

Domain	Task (DV)	OC phase		OC Class	
		Active	Inactive	Anti-Androgenic	Androgenic
**Verbal M(SD)**	List *(words ^a^)*	30.49(3.32)	30.31(2.88)	30.53(3.29)	30.28(2.92)
	List Recall *(words ^a^)*	11.20(1.05)	10.91(1.42)	10.97(1.38)	11.14(1.12)
**Visuospatial M(SD)**	Shapes *(errors ^b^)*	23.54(29.53)	25.09(31.19)	30.85(33.97)	18.14(24.00)
	Maze *(errors ^b^)*	36.51(14.64)	39.46(11.03)	40.76(12.04)	35.36(13.39)
	Maze Recall *(errors ^b^)*	4.69(2.97)	4.94(3.29)	5.91(3.54)	3.78(2.26)
**Attention & Working Memory M(SE)**	One Back *(accuracy ^c^)*	1.346(0.019)	1.365(0.024)	1.350(0.022)	1.361(0.022)
	Two Back *(accuracy ^c^)*	1.299(0.027)	1.342(0.023)	1.329(0.026)	1.312(0.024)
**Executive Function M(SE)**	Set Shifting *(accuracy ^c^)*	1.239(0.008)	1.245(0.008)	1.244(0.008)	1.239(0.008)
**Psychomotor M(SE)**	Identification *(speed ^d^)*	2.693(0.011)	2.698(0.013)	2.702(0.014)	2.689(0.009)
	Chase Test *(moves/sec)*	1.521(0.046)	1.582(0.038)	1.532(0.048)	1.569(0.035)
**Social-Emotional M(SE)**	Social-Emotional *(accuracy ^c^)*	1.151(0.029)	1.132(0.027)	1.078(0.032)	1.202(0.019)

^a^ Words = Total number of words correct; maximum score 36 for List, maximum score 12 for List Recall; ^b^ Errors = Total number of errors made; no maximum for any task; ^c^ Accuracy = Arcsine of the square root of accuracy score; ^d^ Speed = Log_10_(speed in milliseconds).

**Table 5:** Results of MIXED/GLIMMIX procedures models for each task score.

Domain	Task (DV)	Oral Contraceptive Phase ‡				Oral Contraceptive Class †			
		B	SE	P	FDR(P)	B	SE	P	FDR(P)
**Verbal**	List *(words ^a^)*	0.006	0.043	0.92	0.92	0.008	0.043	0.88	0.88
	List Recall * (words ^a^)*	0.026	0.072	0.78	0.87	−0.015	0.072	0.87	0.88
**Visuospatial**	*Shapes (errors*^b^)	−0.1301	0.2245	0.57	0.87	0.6605	0.4109	0.19	0.43
	Maze (errors ^b^)	−0.0913	0.0532	0.10	0.59	0.1588	0.1012	0.13	0.39
	**Maze Recall** (*errors ^b^*)	−0.0459	0.1378	0.74	0.87	**0.4358**	**0.1548**	**0.008**	**0.04**
**Executive Function**	Set Shifting (*accuracy ^c^*)	−0.006	0.009	0.54	0.87	0.005	0.014	0.72	0.88
**Psychomotor**	Identification (*speed ^d^*)	−0.004	0.011	0.69	0.87	0.013	0.021	0.56	0.88
	Chase Test (*moves/sec ^e^*)	−0.061	0.039	0.13	0.59	−0.037	0.074	0.62	0.88
**Social-Emotional**	**Social-Emotional** (*accuracy ^f^*)	0.1076	0.1164	0.36	0.87	**−0.6346**	**0.2444**	**0.01**	**0.04**

^a^ Words = Total number of words correct; maximum score 36 for List, maximum score 12 for List Recall; fitted as count variables with Poisson error distribution and log link; ^b^ Errors = Total number of errors made; no maximum for any task; fitted with Poisson error distribution as count variables with log link; ^c^ Set shifting accuracy = Arcsine of the square root of accuracy score; fitted with normal error distribution (Shapiro–Wilks *p* = 0.15); ^d^ Card identification speed task (log_10_ (speed in milliseconds)); fitted with normal error distribution (Shapiro–Wilks *p* = 0.10); ^e^ Chase test: fitted with normal error distribution (Shapiro–Wilks *p* = 0.71); ^f^ Social-emotional task accuracy; fitted as accuracy proportion with beta error distribution and logit link. **‡** Active phase vs. inactive phase. **†** Anti-androgenic class vs. androgenic. FDR = False discovery rate. Bolded results for *p* < 0.05.

## References

[1] Mazza D., Harrison C., Taft A., Brijnath B., Britt H., Hobbs M., Stewart K., Hussainy S. (2012). Current contraceptive management in Australian general practice: An analysis of BEACH data. Med. J. Aust..

[2] Skovlund C.W., Morch L.S., Kessing L.V., Lidegaard O. (2016). Association of hormonal contraception with depression. JAMA Psychiatry.

[3] Warren A.M., Gurvich C., Worsley R., Kulkarni J. (2014). A systematic review of the impact of oral contraceptives on cognition. Contraception.

[4] Sherwin B.B. (2003). Estrogen and cognitive functioning in women. Endocr. Rev..

[5] Gasbarri A., Pompili A., Clotide Tavares M., Tomaz C. (2009). Estrogen and cognitive functions. Expert Rev. Endocrinol. Metab..

[6] Singh M., Su C. (2013). Progesterone and neuroprotection. Horm. Behav..

[7] Hampson E. (2018). Regulation of cognitive function by androgens and estrogens. Curr. Opin. Behav. Sci..

[8] Luine V., Frankfurt M. (2020). Estrogenic regulation of memory: The first 50 years. Horm. Behav..

[9] Lezak M.D. (2012). Neuropsychological Assessment.

[10] Sherwin B.B. (2012). Estrogen and cognitive functioning in women: Lessons we have learned. Behav. Neurosci..

[11] Andreano J.M., Cahill L. (2009). Sex influences on the neurobiology of learning and memory. Learn. Mem..

[12] Hoffmann H., Kessler H., Eppel T., Rukavina S., Traue H.C. (2010). Expression intensity, gender and facial emotion recognition: Women recognize only subtle facial emotions better than men. Acta Psychol..

[13] Hyde J.S. (2016). Sex and cognition: Gender and cognitive functions. Curr. Opin. Neurobiol..

[14] Buckwalter J.G., Stanczyk F.Z., McCleary C.A., Bluestein B.W., Buckwalter D.K., Rankin K.P., Chang L., Goodwin T.M. (1999). Pregnancy, the postpartum, and steroid hormones: Effects on cognition and mood. Psychoneuroendocrinology.

[15] Henry J.D., Rendell P.G. (2007). A review of the impact of pregnancy on memory function. J. Clin. Exp. Neuropsychol..

[16] Hogervorst E., Bandelow S. (2010). Sex steroids to maintain cognitive function in women after the menopause: A meta-analyses of treatment trials. Maturitas.

[17] Hampson E. (1990). Variations in sex-related cognitive abilities across the menstrual cycle. Brain Cogn..

[18] Hausmann M., Slabbekoorn D., Van Goozen S.H.M., Cohen-Kettenis P.T., Güntürkün O. (2000). Sex hormones affect spatial abilities during the menstrual cycle. Behav. Neurosci..

[19] Derntl B., Kryspin-Exner I., Fernbach E., Moser E., Habel U. (2008). Emotion recognition accuracy in healthy young females is associated with cycle phase. Horm. Behav..

[20] Sundstrom Poromaa I., Gingnell M. (2014). Menstrual cycle influence on cognitive function and emotion processing-from a reproductive perspective. Front. Neurosci..

[21] Manthey D., Behl C. (2006). From structural biochemistry to expression profiling: Neuroprotective activities of estrogen. Neuroscience.

[22] Hara Y., Waters E.M., McEwen B.S., Morrison J.H. (2015). Estrogen effects on cognitive and synaptic health over the lifecourse. Physiol. Rev..

[23] Wierman M.E. (2007). Sex steroid effects at target tissues: Mechanisms of action. Adv. Physiol. Educ..

[24] Reddy D.S. (2010). Neurosteroids: Endogenous role in the human brain and therapeutic potentials. Prog. Brain Res..

[25] Shulman L.P. (2011). The state of hormonal contraception today: Benefits and risks of hormonal contraceptives: Combined estrogen and progestin contraceptives. Am. J. Obstet. Gynecol..

[26] Schindler A.E., Campagnoli C., Druckmann R., Huber J.C., Pasqualini J.R., Schweppe K.W., Thijssen J.H.H. (2003). Classification and pharmacology of progestins. Maturitas.

[27] Guerra J.A., López-Muñoz F., Álamo C. (2013). Progestins in combined contraceptives. J. Exp. Clin. Med..

[28] Montoya E.R., Bos P.A. (2017). How oral contraceptives impact social-emotional behavior and brain function. Trends Cogn. Sci..

[29] Gogos A. (2013). Natural and synthetic sex hormones: Effects on higher-order cognitive function and prepulse inhibition. Biol. Psychol..

[30] Mordecai K.L., Rubin L.H., Maki P.M. (2008). Effects of menstrual cycle phase and oral contraceptive use on verbal memory. Horm. Behav..

[31] Maki P.M., Rich J.B., Rosenbaum R.S. (2002). Implicit memory varies across the menstrual cycle: Estrogen effects in young women. Neuropsychologia.

[32] Beltz A.M., Hampson E., Berenbaum S.A. (2015). Oral contraceptives and cognition: A role for ethinyl estradiol. Horm. Behav..

[33] Islam F., Sparkes C., Roodenrys S., Astheimer L. (2008). Short-term changes in endogenous estrogen levels and consumption of soy isoflavones affect working and verbal memory in young adult females. Nutr. Neurosci..

[34] Mihalik J.P., Ondrak K.S., Guskiewicz K.M., McMurray R.G. (2009). The effects of menstrual cycle phase on clinical measures of concussion in healthy college-aged females. J. Sci. Med. Sport/Sports Med. Aust..

[35] Rosenberg L., Park S. (2002). Verbal and spatial functions across the menstrual cycle in healthy young women. Psychoneuroendocrinology.

[36] Griksiene R., Ruksenas O. (2011). Effects of hormonal contraceptives on mental rotation and verbal fluency. Psychoneuroendocrinology.

[37] Wharton W., Hirshman E., Merritt P., Doyle L., Paris S., Gleason C. (2008). Oral contraceptives and androgenicity: Influences on visuospatial task performance in younger individuals. Exp. Clin. Psychopharmacol..

[38] Kirschbaum C., Kudielka B.M., Gaab J., Schommer N.C., Hellhammer D.H. (1999). Impact of gender, menstrual cycle phase, and oral contraceptives on the activity of the hypothalamus-pituitary-adrenal axis. Psychosom. Med..

[39] Rohleder N., Wolf J.M., Piel M., Kirschbaum C. (2003). Impact of oral contraceptive use on glucocorticoid sensitivity of pro-inflammatory cytokine production after psychosocial stress. Psychoneuroendocrinology.

[40] Mordecai K.L., Rubin L.H., Eatough E., Sundermann E., Drogos L., Savarese A., Maki P.M. (2017). Cortisol reactivity and emotional memory after psychosocial stress in oral contraceptive users. J. Neurosci. Res..

[41] Hamstra D.A., De Rover M., De Rijk R.H., Van der Does W. (2014). Oral contraceptives may alter the detection of emotions in facial expressions. Eur. Neuropsychopharmacol. J. Eur. Coll. Neuropsychopharmacol..

[42] Hamstra D.A., de Kloet E.R., van Hemert A.M., de Rijk R.H., Van der Does A.J. (2015). Mineralocorticoid receptor haplotype, oral contraceptives and emotional information processing. Neuroscience.

[43] Pahnke R., Mau-Moeller A., Junge M., Wendt J., Weymar M., Hamm A.O., Lischke A. (2019). Oral contraceptives impair complex emotion recognition in healthy women. Front. Neurosci..

[44] Radke S., Derntl B. (2016). Affective responsiveness is influenced by intake of oral contraceptives. Eur. Neuropsychopharmacol..

[45] Moos R.H. (1968). The development of a menstrual distress questionnaire. Psychosom. Med..

[46] Beck A.T., Ward C.H., Mendelson M., Mock J., Erbaugh J. (1961). An inventory for measuring depression. Arch. Gen. Psychiatry.

[47] Eaton W.W., Smith C., Ybarra M., Muntaner C., Tien A., Maruish M.E. (2004). Centre for Epidemiological Studies Depression Scale: Review and Revision (CESD and CESD-R). The Use of Psychological Testing for Treatment Planning and Outcomes Assessment: Instruments for Adults.

[48] McNair D.M., Lorr M., Droppelman L.F. (1981). Profile of Mood States.

[49] Pietrzak R., Maruff P., Mayes L., Roman S., Sosa J., Snyder P. (2008). An examination of the construct validity and factor structure of the groton maze learning test, a new measure of spatial working memory, learning efficiency, and error monitoring. Arch. Clin. Neuropsychol..

[50] Collie A., Darekar A., Weissgerber G., Toh M.K., Snyder P.J., Maruff P., Huggins J.P. (2007). Cognitive testing in early-phase clinical trials: Development of a rapid computerized test battery and application in a simulated Phase I study. Contemp. Clin. Trials.

[51] Falleti M.G., Maruff P., Collie A., Darby D.G. (2006). Practice effects associated with the repeated assessment of cognitive function using the CogState battery at 10-minute, one week and one month test-retest intervals. J. Clin. Exp. Neuropsychol..

[52] Sitruk-Ware R., Nath A. (2010). The use of newer progestins for contraception. Contraception.

[53] Moffat S.D., Hampson E. (1996). A curvilinear relationship between testosterone and spatial cognition in humans: Possible influence of hand preference. Psychoneuroendocrinology.

[54] Hua J.T., Hildreth K.L., Pelak V.S. (2016). Effects of testosterone therapy on cognitive function in aging: A systematic review. Cogn. Behav. Neurol. Off. J. Soc. Behav. Cogn. Neurol..

[55] Wisniewski A.B., Nguyen T.T., Dobs A.S. (2002). Evaluation of high-dose estrogen and high-dose estrogen plus methyltestosterone treatment on cognitive task performance in postmenopausal women. Horm. Res..

[56] Picot M., Billard J.M., Dombret C., Albac C., Karameh N., Daumas S., Hardin-Pouzet H., Mhaouty-Kodja S. (2016). Neural androgen receptor deletion impairs the temporal processing of objects and hippocampal CA1-dependent mechanisms. PLoS ONE.

[57] Hampson E., Vananders S., Mullin L. (2006). A female advantage in the recognition of emotional facial expressions: Test of an evolutionary hypothesis. Evol. Hum. Behav..

[58] Osório F.L., de Paula Cassis J.M., Machado de Sousa J.P., Poli-Neto O., Martín-Santos R. (2018). Sex hormones and processing of facial expressions of emotion: A systematic literature review. Front. Psychol..

[59] Marecková K., Perrin J.S., Nawaz Khan I., Lawrence C., Dickie E., McQuiggan D.A., Paus T., Consortium I. (2014). Hormonal contraceptives, menstrual cycle and brain response to faces. Soc. Cogn. Affect. Neurosci..

[60] Pletzer B., Kronbichler M., Kerschbaum H. (2015). Differential effects of androgenic and anti-androgenic progestins on fusiform and frontal gray matter volume and face recognition performance. Brain Res..

[61] Hermans E.J., Ramsey N.F., van Honk J. (2008). Exogenous testosterone enhances responsiveness to social threat in the neural circuitry of social aggression in humans. Biol. Psychiatry.

[62] van Wingen G.A., Zylicz S.A., Pieters S., Mattern C., Verkes R.J., Buitelaar J.K., Fernandez G. (2009). Testosterone increases amygdala reactivity in middle-aged women to a young adulthood level. Neuropsychopharmacol. Off. Publ. Am. Coll. Neuropsychopharmacol..

[63] Bos P.A., van Honk J., Ramsey N.F., Stein D.J., Hermans E.J. (2013). Testosterone administration in women increases amygdala responses to fearful and happy faces. Psychoneuroendocrinology.

